# Sensitive reverse transcription-digital droplet PCR-based discrimination of hepatitis delta virus RNAs reveals differential effects of interferon-α on viral RNA species

**DOI:** 10.1099/jgv.0.002324

**Published:** 2026-10-07

**Authors:** Caroline Pons, Emilie Charles, Cassandre Authier, Elise Chabot, Roxanne Fouillé, Faustine Bernardin, Claudie Eber, Léa Corbet, Maud Michelet, Marine Oudot, Charlotte Bach, Michel Rivoire, Guillaume Passot, Thomas F. Baumert, Karim Majzoub, Eric Huntzinger, Bertrand Seraphin, David Durantel, Eloi R. Verrier, Julie Lucifora

**Affiliations:** 1 CIRI, Centre International de Recherche en Infectiologie, Univ Lyon, Inserm, U1111,, Université Claude Bernard Lyon 1, CNRS, UMR5308, ENS de Lyon, F-69007, Lyon, France; 2 Institute for Translational Medicine and Liver Disease, Université de Strasbourg, Inserm, UMRS 1110, Strasbourg, France; 3 INSERM, U1052, Cancer Research Center of Lyon (CRCL), University of Lyon (UCBL1), CNRS UMR_5286, Centre Léon Bérard, Lyon, France; 4 Centre Léon Bérard (CLB), INSERM, U1032, Lyon, France; 5 Service de chirurgie générale et oncologique, Hôpital Lyon Sud, Hospices Civils de Lyon Et CICLY, EA3738, Université Lyon 1, Lyon, France; 6 Pôle Hépato-digestif, Service d'Hépato-gastroentérologie, Hôpitaux Universitaires de Strasbourg, Strasbourg, France; 7 Institut universitaire de France (IUF), Paris, France; 8 Institut de Génétique Moléculaire de Montpellier (IGMM), Université de Montpellier, CNRS UMR5535, Montpellier, France; 9 IGBMC, Institut de Génétique et de Biologie Moléculaire et Cellulaire, CNRS UMR 7104, Inserm U 1258, Université de Strasbourg, Illkirch, France

**Keywords:** antivirals, hepatitis delta virus (HDV), interferon (IFN), interferon-stimulated gene (ISG), reverse transcription digital droplet PCR (RT-ddPCR)

## Abstract

Hepatitis delta virus (HDV) replication relies on the production of distinct viral RNA species, including genomic (HDV-G), antigenomic (HDV-AG) and mRNAs (HDV mRNAs), which differ in abundance and function. However, the lack of sensitive and quantitative approaches allowing their simultaneous discrimination has limited the precise analysis of HDV RNA dynamics, particularly under antiviral conditions. Here, we combined nascent RNA and RNA stability assays with a novel reverse transcription digital droplet PCR (RT-ddPCR) approach enabling strand-specific quantification of HDV RNAs. Using this methodology, we confirmed the previously described kinetics of viral RNA production and assessed the ratio of each RNA species in HDV-infected cells. Specific small interfering RNA (siRNA) treatment demonstrated the specificity of our assay in discriminating HDV RNA species. Next, applying this technique to the study of virus-host interactions, we showed that IFN-α treatment decreases the synthesis of HDV-G and may additionally accelerate HDV-AG and HDV-G decay, while HDV mRNA stability remained unaffected in infected HuH7.5-Na+-taurocholate cotransporting polypeptide (NTCP) cells. Immunoprecipitation assays coupled to strand-specific RT-ddPCR revealed no difference in the association of cellular RNA polymerase II (Pol II) with HDV-G and HDV-AG. In parallel, transcriptomic analyses of differentiated HepaRG and HuH7.5-NTCP cells following IFN-α treatment identified IFN-stimulated gene candidates with potential anti-HDV activity. Among them, we showed that HELZ2 overexpression does not affect HDV RNA stability but interferes with HDV-AG and HDV-G RNA synthesis. Altogether, this RT-ddPCR-based approach provides a versatile tool to investigate the effects of current and emerging antiviral strategies on individual HDV RNA species.

## Data Availability

The data presented in this manuscript are available through the corresponding author (Julie Lucifora) upon reasonable request. Next-generation sequencing was performed by the Biomedical Sequencing Facility at CeMM Research Center for Molecular Medicine of the Austrian Academy of Sciences. RNA-seq data presented in this manuscript are accessible through Gene Expression Omnibus (GSE288203).

## Introduction

Hepatitis delta virus (HDV) is a defective virus that hijacks the envelope surface glycoproteins of hepatitis B virus (HBV) to spread into hepatocytes. In 2022, the World Health Organization estimated that 254 million people were chronically infected with HBV worldwide, among whom ~12 million were chronically co-infected with HDV [1]. HDV/HBV co-infected patients suffer from the most aggressive form of chronic viral hepatitis, frequently leading to severe liver diseases such as cirrhosis, liver decompensation and hepatocellular carcinoma. HDV circulates as a ribonucleoprotein (RNP) composed of a 1.7 kb circular single-stranded negative RNA genome forming a ‘quasi’ double-stranded structure, associated with the small and large hepatitis delta antigens (S-HDAg and L-HDAg) (see [2] for a comprehensive review). In the nucleus of infected cells, the incoming HDV genome (HDV-G) serves as a template for S-HDAg mRNA transcription as well as for the synthesis of the antigenomic RNA (HDV-AG), by hijacking the cellular DNA-dependent RNA polymerase II (Pol II) and/or RNA polymerase I or III [2]. HDV-G and HDV-AG are produced through a rolling-circle replication mechanism. Ribozyme activities embedded in both RNAs mediate the cleavage of multimeric intermediates into unit-length HDV-G or HDV-AG molecules [2]. A fraction of HDV-AG is subsequently edited by ADAR1, resulting in the expression of the large antigen (L-HDAg). While S-HDAg is essential for HDV RNA replication and mRNA transcription, L-HDAg is required for virion assembly in the Golgi apparatus [2]. HDV replication therefore relies on the coordinated production of distinct viral RNA species that differ in abundance and function. Pegylated IFN-α (PEG-IFN-α) has long remained the first-line treatment for chronic HDV infection. Although its use is associated with significant side effects, around 25% of patients achieved a sustained HDV-negative viraemia [3, 4]. Moreover, recent clinical trials have demonstrated an added benefit when PEG-IFN-α is combined with other antiviral compounds such as bulevirtide or lonafarnib [5–7], supporting its continued relevance in HDV therapy. The modes of action of PEG-IFN-α are thought to be multiple: stimulation of the immune system and direct effects on virus replication in hepatocytes. Type I IFNs initiate signalling through the IFN-α/β receptor (IFNAR), activating the Jak–STAT pathway and inducing the expression of hundreds of IFN-stimulated genes (ISGs) [8]. Several ISG-encoded effectors have been shown to inhibit HBV replication in hepatocytes [9]. For instance, MX2 decreases HBV RNA synthesis and potently restricts HBV infection by impairing cccDNA formation [10]. Early studies using transfection assays or 293 cells allowing HDV replication through genome integration reported *in vitro* resistance of HDV to IFN-α treatments [11–13]. Later studies suggested, however, that IFN-α impedes early steps of viral infection [14, 15] and cell-to-cell-mediated spread [16, 17]. In addition, we reported that repeated IFN-α treatments applied after infection in differentiated, non-dividing hepatocyte models result in reduced intracellular HDV RNA and protein levels, as well as decreased infectivity of progeny virions [18–20]. Despite these observations, the molecular mechanisms underlying the direct antiviral activity of IFN-α against HDV in hepatocytes remain largely unknown. To date, only proviral ISG-encoded factors, including ADAR1 [21, 22], MOV10 [23] and nuclear body-associated proteins such as PML and Sp100 [24, 25] have been described. Previous studies have analysed HDV RNA accumulation under inhibitory conditions but were based on Northern blot [12] or Reverse Transcription quantitative Polymerase Chain Reaction (RT-qPCR) [26] analyses, which are comparatively less sensitive or rely on standard curves for quantification, potentially introducing bias in absolute measurements of HDV RNA species. In this study, we developed a sensitive RT-ddPCR-based approach enabling the discrimination of individual HDV RNA species and used this methodology to assess the effects of IFN-based treatments on HDV RNA synthesis and stability.

We first showed that total HDV RNAs can be accurately quantified using a digital droplet PCR (ddPCR) assay compared to regular RT-qPCR assays (Fig. 1a) and next developed a robust method to quantify each HDV RNA species using a combination of specific retro-transcription reactions and probes (Fig. 1b). We confirmed previously reported data from transfection assays [27, 28] by demonstrating a one log_10_ difference between HDV mRNA and HDV-AG levels and a one log_10_ difference between HDV-AG and HDV-G levels in dHepaRG cells co-infected with HBVHDV, with HDV-G being the most abundant HDV RNA species (Fig. 1c). As a control to demonstrate the specificity of the assay, we used cells transduced with adeno-associated virus (AAV) vectors allowing only the production of S-HDAg mRNA and showed that neither HDV-G nor HDV-AG is detected (Fig. 1c). This new assay was applied to kinetic experiments in HBV/HDV co-infected dHepaRG cells and we showed that very low HDV-AG and HDV mRNA levels could be quantified 24 h post-inoculation with HDV (Fig. 1d, e). Both HDV RNA species displayed an amplification profile with similar kinetics over 20 h to reach HDV-G levels that were detected 24 h post-infection and remained stable until around 52 h post-inoculation (Fig. 1d, e). Amplification of both HDV-G and HDV-AG was then observed, whereas HDV mRNA levels reached a plateau (Fig. 1d, e). These data confirmed previous observations and support that HDV mRNA and HDV-AG are produced from incoming HDV-G and that S-HDAg (and therefore HDV mRNA) and HDV-AG are required for productive HDV-G amplification [27, 29, 30]. Moreover, these data demonstrate that quantification of total HDV RNA does not allow discrimination of variations in HDV-AG RNA and mRNA, as genomic RNA levels predominate (Fig. 1d). Performance of the assay was functionally validated using siRNA specifically targeting HDV mRNA (and theoretically also HDV-AG) sequences in short kinetic assays. The reduction of all HDV RNA species with siHDV at late time points (56 h and 72 h) was expected (Fig. 1f), given their strong interdependence during the HDV replication cycle [2]. At early time point (32 h and 48 h), we did not observe any differences in the levels of HDV-G, reflecting the predominant contribution of input viral RNA at these stages (Fig. 1d). In contrast, levels of HDV mRNAs and HDV-AG, which are newly synthesized (Fig. 1d), were reduced by 60% as early as 32 h post-infection upon treatment with siHDV (Fig. 1f). The early decrease in HDV-AG was, however, unexpected, as this RNA species is generally considered to be predominantly nuclear and therefore less accessible to siRNA-mediated silencing [31]. This effect may reflect an indirect and rapid impact on ongoing HDV RNA synthesis following siRNA treatment rather than direct targeting of HDV-AG.

**Fig. 1. F1:**
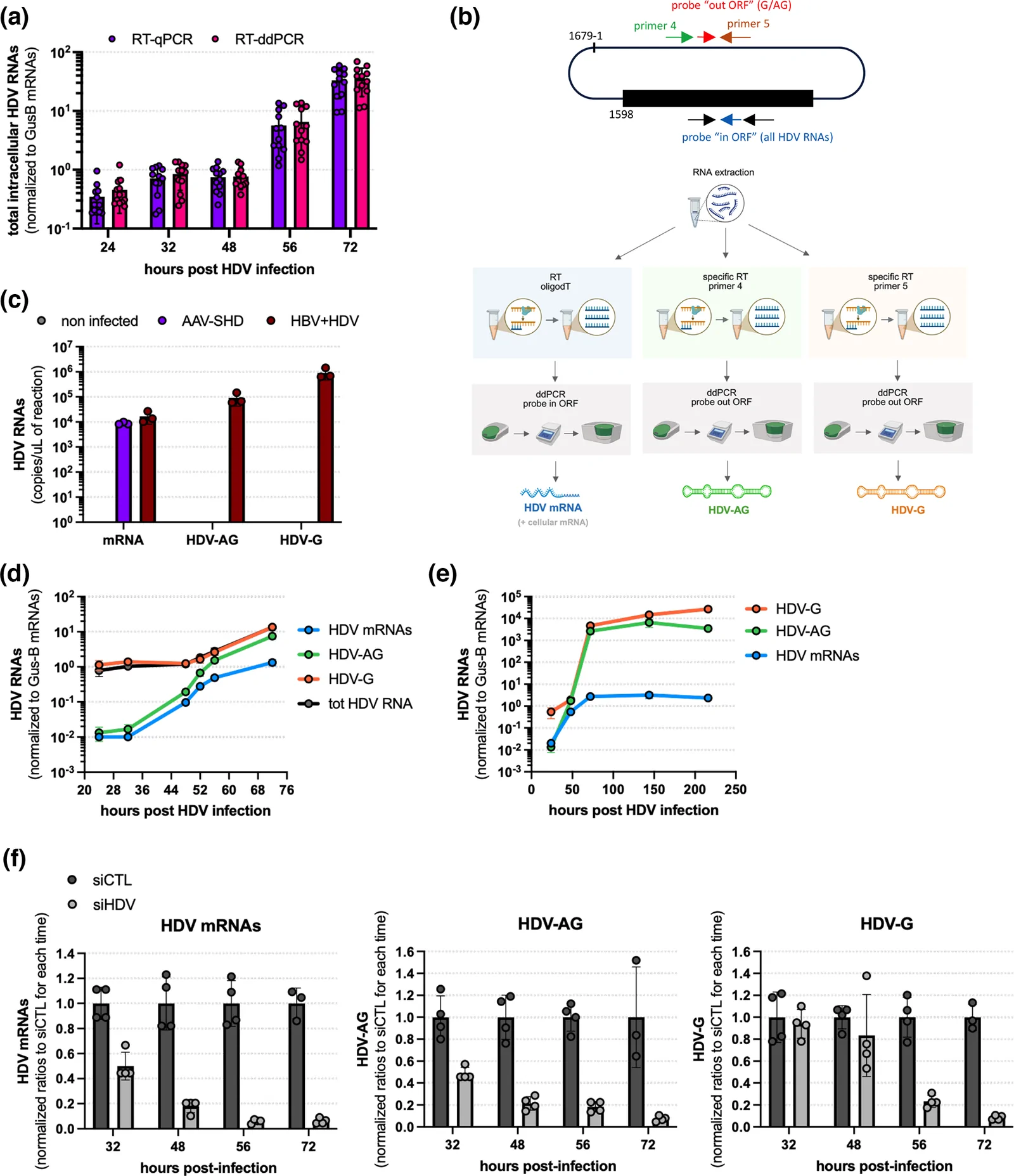
Quantification of HDV RNA species by a specific RT-ddPCR assay. (**a**) HuH7-NTCP cells were infected with HDV, and levels of total intracellular HDV RNAs were determined at the indicated times by RT-qPCR or RT-ddPCR analyses. Results are the mean±sd of three independent experiments, each performed with four distinct infected wells (biological replicates) and subsequent analyses. Dots represent each biological replicate. (**b**) Schematic representation of the assay to distinguish between HDV RNA species; figure created with BioRender, Lucifora, J. (2025) https://BioRender.com/hr4xcz1. (**c**) dHepaRG cells were infected with HBV and HDV or transduced with AAV-small HDV protein (SHD). Eight days later, cells were collected, RNAs were extracted, and levels of HDV mRNA, HDV-AG and HDV-G were quantified by RT-ddPCR analyses. Results are the mean±sd of three distinct infected wells (biological replicates) and subsequent analyses from one experiment. Dots represent each biological replicate. (**d, e**) dHepaRG cells were infected with HBV and HDV. At the indicated time post-infection, cells were collected, RNAs were extracted, and levels of HDV mRNA, HDV-AG, HDV-G and total HDV RNAs were quantified by RT-ddPCR analyses. (**d**) and (**e**) are two independent experiments, each performed with three distinct infected wells (biological replicates) and subsequent analyses. (**f**) HuH7-NTCP cells were transfected with control siRNA (siCTL) (25 nM final) or siRNA targeting HDV (siHDV) (25 nM final, mix of two siRNAs theoretically directed against the HDV mRNAs and HDV-AG) 24 h before infection with HDV. At the indicated time post-infection, levels of HDV mRNA, HDV-AG and HDV-G were quantified by RT-ddPCR analyses. Results are the mean±sd of three to four distinct infected wells (biological replicates) and subsequent analyses. Dots represent each biological replicate.

Both HDV-G and HDV-AG levels were shown to decrease upon PEG-IFN-α treatment in humanized liver mice, although some HDV strains appeared to be less sensitive than others [26, 32]. Here, we further characterized the antiviral effect of IFN-α on HDV RNA species by performing different assays coupled to our newly developed RT-ddPCR assay. We first consolidated our previous observations that several treatments with high doses of IFN-α are needed to significantly decrease intracellular HDV RNA levels in resting/non-dividing cells [18, 19] using DMSO-treated HuH7.5-NTCP cells (dHuH7.5-NTCP), an alternative *in vitro* model for resting hepatocytes [33]. In this model, where HDV infection does not induce endogenous type-I IFN production itself [15], we observed an 80% reduction of intracellular HDV RNA levels upon IFN-α treatment from day 3 to day 10 post-infection (Fig. 2a). A similar antiviral effect of IFN-α could be observed in HDV mono-infected dHuH7.5-NTCP cells with a shorter treatment duration applied early after infection (Fig. 2b) but not with shorter treatment duration applied at later time points (Fig. 2c), suggesting that prolonged treatment with IFN-α is necessary to observe an antiviral effect in *in vitro* resting cells. Of note, it has been reported that IFN-α inhibits HDV cell-division-mediated spread, while replication in resting cells remained unaffected [16]. The discrepancy between this study and our results could be attributed to the difference in the frequency and treatment dose used in the different studies, and observations are not mutually exclusive, as the impact of IFN-α on HDV replication during cell division is attributed to the breakdown of the nuclear membrane, which releases viral RNA into the cytoplasm, where it becomes more accessible to ISG-encoded antiviral effectors (reviewed in [34]). This is consistent with a moderate effect of IFN-α in resting cells, where most but not all viral RNAs remain protected within the nucleus. Given that most hepatocytes are quiescent and that PEG-IFN-α is repeatedly administered to patients, it is likely that at least part of the antiviral effect observed in patients could be attributed to a direct inhibition of viral replication in resting cells. We next investigated the effect of IFN-α on HDV RNA species stability by performing RNA stability coupled to our newly developed RT-ddPCR assay. HDV-infected HuH7.5-NTCP cells were treated or not with IFN-α for 4 days and incubated with bromo-uridine (BrU) to label nascent RNAs for 24 h (pulse). After extensive washes, cells were further cultured in the presence of IFN-α and harvested at different times (chase). Labelled RNAs were purified by immunoprecipitation and quantified by RT-ddPCR analyses. Consistent with previous studies suggesting that HDV mRNA is less stable than HDV-G and HDV-AG [29, 35, 36], we observed a shorter half-life for HDV mRNA. Our data further provide a quantitative assessment of these differences; in the non-treated condition, we determined a half-life of around 7 h for HDV mRNA, whereas the half-lives of HDV-AG and HDV-G were >24 h (Fig. 2d), in accordance with their rod-like structure, which may protect from nuclease activity. We observed that IFN-α treatments resulted in the fastest decay of BrU-labelled HDV-AG (half-life of around 12 h) and HDV-G (half-life of around 16 h) but had no effect on BrU-labelled HDV mRNA decay (Fig. 2d). Although modest, these data suggest that enhanced RNA decay may contribute to the antiviral activity of IFN-α. These observations also suggest a sequence-independent activity and the involvement of IFN-induced nucleases capable of targeting either the pseudo-double-stranded RNA structure of HDV or the single-stranded HDV-G/HDV-AG multimers generated through the rolling circle replication mechanism. Of note, IFN-α treatments did not affect the stability of the cellular GusB mRNAs we used as a reference gene throughout our study (Fig. 2d). To determine if IFN-α might also affect HDV RNA synthesis, we performed nascent RNA assays. Since the levels of HDV mRNA, HDV-AG and HDV-G RNAs are tightly linked [2][Fig F1], analyses were performed when no significant difference in the levels of total HDV RNAs with IFN-α treatment could be observed yet (Fig. 1c). Three days after IFN-α treatment, cells were incubated with BrU for 2 h and labelled nascent RNAs were quantified by RT-qPCR (Fig. 2e, left panel) and RT-ddPCR. As controls for specificity of the labelling and purification, we used cells not incubated with BrU or cells incubated with BrU and actinomycin D (ActD), a known inhibitor of RNA synthesis [37, 38] (Fig. 2e, middle panel). Using our new RT-ddPCR method, we confirmed the previous observation [12] that HDV-G and HDV-AG levels are weakly sensitive to ActD, whereas HDV and Gus B mRNA production is strongly affected by ActD, which may reflect the difference between the production of HDV mRNA and the replication of HDV-G and HDV-AG through a rolling-circle mechanism. We observed decreases in BrU-labelled HDV-G upon IFN-α treatment (Fig. 2e, right panel), demonstrating that IFN-α treatment first impairs the synthesis of this HDV RNA species. This selective activity indicates that IFN-α may act directly on the HDV replication complex, potentially by interfering with the rolling-circle replication template. However, using immunoprecipitation assays followed by RNA extraction, we did not observe differences in the levels of HDAg or RNA Pol-II associated with HDV-AG and HDV-G (Fig. 2f) or RNA Pol-II associated with nascent (BrU labelled) HDV-AG and HDV-G (Fig. 2g), indicating that these two important drivers of HDV transcription are not initially affected by IFN-α treatment.

**Fig. 2.**
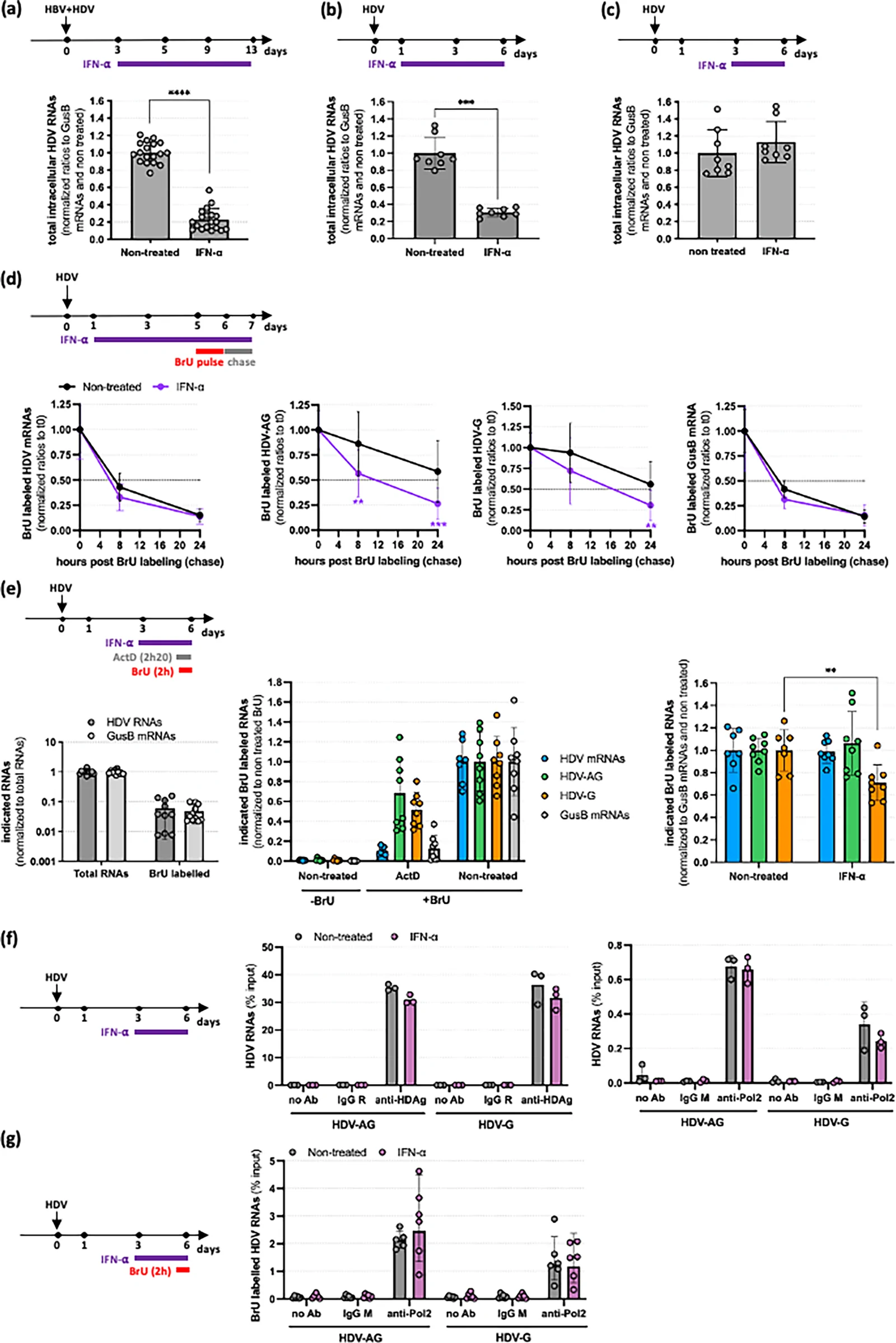
IFN-α treatments differentially affect HDV RNA species. (**a**) dHuH7.5-NTCP cells were co-infected with HBV (500 vge per cell) and HDV (10 vge per cell) and treated or not 3 days later for 10 days with IFN-α (500 IU/mL) as indicated. At 13 days post-infection (dpi), intracellular RNAs were extracted, and the levels of total viral RNAs were analysed by RT-qPCR. Bars are the means±sd of five independent experiments, each performed with three to four distinct infected wells (biological replicates) and subsequent analyses. Dots represent each biological replicate. (**b**) dHuH7.5-NTCP cells were infected with HDV (10 vge per cell) and treated or not 1 day later for 5 days with IFN-α (500 IU/mL) as indicated. At 6 dpi, intracellular RNAs were extracted, and the levels of total HDV RNAs were analysed by RT-qPCR. Bars are the means±sd of two independent experiments, each performed with four distinct infected wells (biological replicates) and subsequent analyses. Dots represent each biological replicate. (**c**) dHuH7.5-NTCP cells were infected with HDV (10 vge per cell) and treated or not 3 day later for 3 days with IFN-α (500 IU/mL) as indicated. At 6 dpi, intracellular RNAs were extracted, and the levels of total HDV RNAs were analysed by RT-qPCR. Bars are the means±sd of two independent experiments, each performed with four distinct infected wells (biological replicates) and subsequent analyses. Dots represent each biological replicate. (**d**) HuH7.5-NTCP cells were infected with HDV (10 vge per cell) and treated or not 1 day later with IFN-α (500 IU/mL) as indicated. At 5 dpi, cells were incubated with BrU for 24 h (pulse). After extensive washes, cells were further cultivated and harvested at the indicated time post-washes (chase). BrU-labelled HDV mRNAs, HDV-G and HDV-AG were quantified by immunoprecipitation followed by RT-ddPCR analyses. Data are the means±sd of five independent experiments, each performed with four distinct infected wells (biological replicates) and subsequent analyses. (**e**) dHuH7.5-NTCP cells were infected with HDV (10 vge per cell) and treated or not 3 day later with IFN-α (500 IU/mL) as indicated. At 6 dpi, cells were incubated or not with BrU for 2 h. As a control, some cells were treated with ActD 20 min before BrU was added to the culture media and kept for the 2 h incubation with BrU. Total intracellular and BrU-labelled HDV RNAs and GusB mRNAs were quantified by RT-qPCR analyses (left panel). BrU-labelled HDV mRNAs, HDV-G, HDV-AG and GusB mRNAs were quantified by immunoprecipitation followed by RT-ddPCR analyses (middle and right panels). Data are the means±sd of two to four independent experiments, each performed with three to four distinct infected wells (biological replicates) and subsequent analyses. Dots represent each biological replicate. (**f**) dHuH7.5-NTCP cells were infected with HDV (10 vge per cell) and treated or not 3 day later with IFN-α (500 IU/mL) for 3 days. Cells were cross-linked before lysis and immunoprecipitation with the indicated antibodies. RNA associated with the immunoprecipitated proteins was purified, and levels of HDV-AG and HDV-G were assessed by specific RT-ddPCR. Results are the mean of three distinct infected wells (biological replicates) and subsequent analyses from one representative experiment. Dots represent each biological replicate. (**g**) dHuH7.5-NTCP cells were infected with HDV (10 vge per cell) and treated or not 3 day later with IFN-α (500 IU/mL) for 3 days. At 6 dpi, cells were incubated with BrU for 2 h, washed and cross-linked before lysis, nucleus purification and immunoprecipitation with the indicated antibodies. RNA associated with the immunoprecipitated proteins was purified, and levels of total HDV RNA were assessed by specific RT-qPCR. BrU-labelled RNA was purified from immunoprecipiatation (IP) eluate, and levels of HDV-AG and HDV-G were assessed by specific RT-ddPCR. Results are the mean±sd of two independent experiments, each performed with three distinct infected wells (biological replicates) and subsequent analyses. Dots represent each biological replicate.

This multimodal effect of IFN-α on HDV RNAs is consistent with observations in other viral systems, given the large number of ISGs with antiviral potential that are upregulated upon IFN-α treatment. It is therefore likely that several ISGs act concomitantly to inhibit HDV replication at different steps. To address this, we investigated candidate ISGs that may contribute to this process. We previously observed that IFN-α treatment had no impact on HBV RNA levels in HBV/HDV co-infected dHuH7.5-NTCP cells [33] and therefore hypothesized that different sets of ISGs might be induced upon IFN-α treatment in dHuH7.5-NTCP and dHepaRG cells. To validate this hypothesis, we stimulated these cells with IFN-α for 8 h and analysed gene expression by RNA-seq. Overall, we observed a stronger upregulation of ISGs upon IFN-α stimulation in dHepaRG cells compared to dHuH7.5-NTCP cells (Fig. S1A, available in the online Supplementary Material) and found 150 genes significantly upregulated in both cell lines, whereas 179 and 53 were specifically upregulated in dHepaRG or dHuH7.5-NTCP cells, respectively (Fig. S1A). Since IFN-α treatments lead to decreased levels of intracellular HDV RNAs in both dHepaRG and dHuH7.5-NTCP, we hypothesized that genes encoding antiviral effectors targeting HDV were present among the 150 ISGs commonly upregulated in both cell lines. Based on their potential antiviral effect on RNA regulation and/or RNA viruses, we selected 76 ISGs and confirmed their upregulation using the NanoString nCounter system (Fig. S2). We selected the HELZ2 protein, which has been reported as a restriction factor against dengue virus [39], based on its described exoribonuclease activity, which could have accounted for a potential impact on HDV RNA levels [40]. Its expression profile, upon IFN-α treatment, showed a rapid and similar induction in both dHepaRG and dHuH7.5-NTCP cells, peaking around 8 h (Figs S1B and S2). In line with the MDA5-dependent activation of type I and type III IFN pathways in dHepaRG but not in HuH7.5-NTCP cells upon HDV infection [15, 41], levels of *HELZ2* mRNAs were increased by HDV infection in dHepaRG but not in dHuH7.5-NTCP cells (Fig. S1C). Upregulation of *HELZ2* expression was partially but significantly inhibited in IFN-α-treated, HDV-infected dHepaRG cells compared to IFN-α-treated, non-infected cells (Fig. S1C), supporting previous observations of HDV interfering with ISG upregulation [42, 43]. Interestingly, *HELZ2* expression was not inhibited in IFN-α-treated, HDV-infected dHuH7.5-NTCP cells, correlating with a greater antiviral effect of IFN-α in this model. We next performed gain-of-function assays in different *in vitro* models to determine if *HELZ2* overexpression may affect HDV RNAs. The *HELZ2* gene produces different proteins: HELZ2α, HELZ2β and HELZ2, the latter being produced through the use of a non-canonical translation initiation codon [40]. Decreases in intracellular HDV proteins and RNA levels were observed in HDV-producing HuH7-2C8D cells [44] transduced with lentivectors allowing overexpression of HELZ2β (HA-tagged) compared to cells transduced with the GFP (HA-tagged) control lentivector (Fig. S3A). We confirmed the decrease in the levels of intracellular HDV proteins and RNAs (40% reduction) in HDV infection systems using primary human hepatocytes (PHHs) or HuH7.5-NTCP upon overexpression of HELZ2β (HA-tagged) or HELZ2 (GFP-tagged) (Fig. S3A, B). More precisely, using BrU to label nascent HDV RNAs, we demonstrated that overexpression of HELZ2 ultimately led to the reduction of the synthesis of each type of HDV RNAs in HDV-infected HuH7.5-NTCP cells (Fig. 3a). Of note, we also observed that overexpression of HELZ2 (GFP-tagged) also decreased the levels of rodent deltavirus (RDeV) antigen (RDAg) and woodchuck deltavirus (mmDeV) antigen (mmDAg) in HuH7.5-NTCP cells (Fig. S3C), suggesting a broad antiviral action of HELZ2 against deltavirus replication, including against other *Kolmioviridae* family members. Next, we performed RNA stability assays using BrU to label viral RNAs followed by immunoprecipitation and quantification by RT-ddPCR. We observed no decrease in the half-life of HDV mRNAs, HDV-AG or HDV-G upon overexpression of HELZ2 compared to the Maltose-Binding Protein (MBP) control (Fig. 3b). We next assessed whether HELZ2 overexpression might affect synthesis of HDV RNAs. We observed a decrease in the levels of nascent HDV mRNA, HDV-AG and HDV-G upon overexpression of HELZ2 (Fig. 3a, right panel). However, since levels of the three forms of HDV RNAs are tightly linked during the HDV replication cycle [2], we repeated the nascent RNA assays early after overexpression of HELZ2 when levels of total intracellular HDV RNAs were not affected yet (Fig. 3c, middle panel). We observed that overexpression of HELZ2 (Fig. 3c, left panel) first led to a decrease in the levels of nascent HDV-AG, and even though not significant, we also observed a tendency towards a decrease in the levels of HDV-G (Fig. 3c, right panel). On the contrary, levels of nascent HDV mRNAs were not affected. Altogether, our data suggest that overexpression of HELZ2 interferes with the synthesis of HDV-AG. HELZ2 has been reported to interact with nuclear factors such as PPARα, PPARγ, AHR and SFPQ [39, 45, 46]. Notably, SFPQ has been shown to bind HDV RNA [47] as well as the CTD domain of RNA Pol II [48], where it plays a key role in transcription. We can hypothesize that HELZ2 may interact with the transcription complex critical for HDV replication, thereby disrupting the replication process. This would be consistent with a broad impact of HELZ2 on other deltavirus replication shown for RDeV and mmDeV, as they are expected to share a similar RNA Pol II-dependent replication mechanism [49, 50]. Further studies will be required to define in detail how HELZ2 may exert an antiviral effect and to uncover the complete set of ISGs responsible for the antiviral effect of IFNα on HDV.

**Fig. 3.**
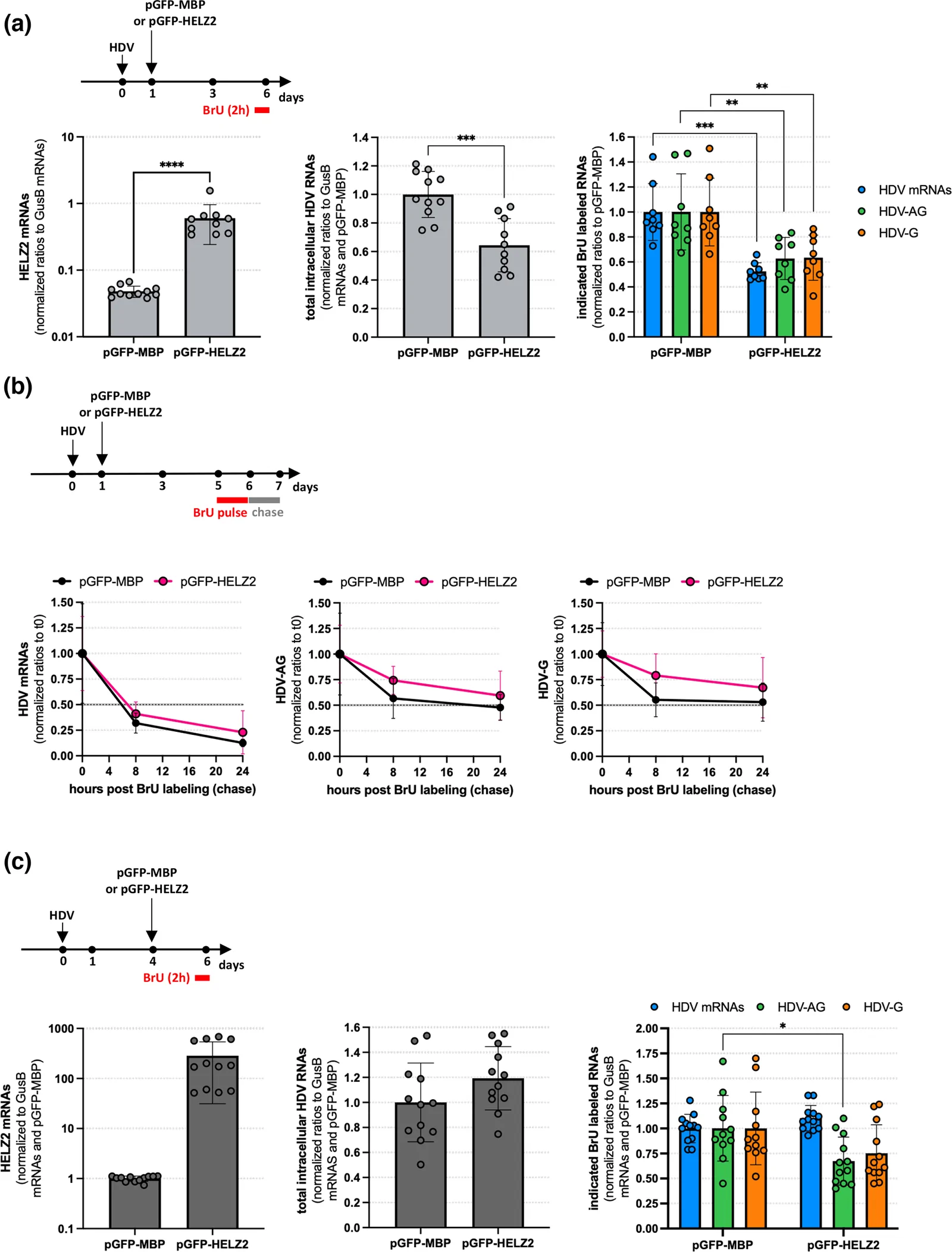
Overexpression of HELZ2 differentially affects HDV RNA species. (**a**) HuH7.5-NTCP cells were infected with HDV (10 vge per cell) and transfected 1 day later with plasmids encoding a GFP-tagged version of HELZ2 or MBP (control protein). At 6 dpi, cells were incubated with BrU for 2 h before intracellular RNA extractions. The levels of *HELZ2* mRNAs and total HDV RNAs were analysed by RT-qPCR (left and middle graphs). BrU-labelled HDV mRNAs, HDV-G and HDV-AG were quantified by immunoprecipitation followed by RT-ddPCR analyses (right graph). Bars are the means±sd of three independent experiments, each performed with three to four distinct infected wells (biological replicates) and subsequent analyses. Dots represent each biological replicate. (**b**) HuH7.5-NTCP cells were seeded and infected with HDV (10 vge per cell) 1 day later. 24 h post-infection, cells were transfected with plasmids encoding a GFP-tagged version of HELZ2 or MBP (control protein). At 5 dpi, cells were incubated with BrU for 24 h (pulse). After extensive washes, cells were further cultivated and harvested at the indicated time (chase). BrU-labelled HDV mRNAs, HDV-G, HDV-AG and GusB mRNAs were quantified by immunoprecipitation followed by RT-ddPCR analyses. Data are the means±sd of two independent experiments, each performed with four distinct infected wells (biological replicates) and subsequent analyses. (**c**) HuH7.5-NTCP cells were seeded and infected with HDV (10 vge per cell) 1 day later. Four dpi, cells were trypsinized and reverse-transfected with plasmids encoding a GFP-tagged version of HELZ2 or MBP (control protein). At 6 dpi, cells were incubated with BrU for 2 h. BrU-labelled HDV mRNAs, HDV-G, HDV-AG and GusB mRNAs were quantified by immunoprecipitation followed by RT-ddPCR analyses. Data are the means±sd of three independent experiments, each performed with four distinct infected wells (biological replicates) and subsequent analyses. Dots represent each biological replicate.

In the future, our HDV strand-specific RT-ddPCR assay will be a versatile tool to dissect the molecular biology of the virus, untangle its complex replication cycle and investigate the effects of emerging antiviral strategies on individual HDV RNA species.

## Supplementary material

10.1099/jgv.0.002324 Fig. S1.

## Abbreviations

AAV: adeno-associated virus
ActD: actinomycin D
BrU: bromo-uridine
ddPCR: digital droplet PCR
dpi: days post-infection
HBV: hepatitis B virus
HDV: hepatitis delta virus
HDV-AG: HDV antigenomic
HDV-G: HDV genomic
IFNAR: IFN-α/β receptor
ISG: IFN-stimulated gene
l-HDAg: large hepatitis delta antigen
mmDAg: woodchuck antigen
mmDeV: woodchuck deltavirus
PEG-IFN-α: Pegylated IFN-α
PHH: primary human hepatocyte
Pol: polymerase
RDAg: rodent antigen
RDeV: rodent deltavirus
RNP: ribonucleoprotein
RT-ddPCR: reverse transcription digital droplet PCR
S-HDAg: small hepatitis delta antigen
