# Supplementary material for: Sensitive reverse transcription-digital droplet PCR-based discrimination of hepatitis delta virus RNAs reveals differential effects of interferon-α on viral RNA species
Source: J Gen Virol. 2026 Oct 7;107(10):002324. doi: 10.1099/jgv.0.002324 (PMC13644934; doi:10.1099/jgv.0.002324)
Supplement: Supplementary file 1 [file jgv-107-02324-s001.pdf]

Sensitive RT-ddPCR-based discrimination of HDV RNAs reveals differential effects of interferon-α on viral RNA species

Caroline Pons[1,*], Emilie Charles[1,*], Cassandre Authier[1,*], Elise Chabot[1], Roxanne Fouillé[1], Faustine Bernardin[1], Claudie Eber[2], Léa Corbet[1], Maud Michelet[3], Marine Oudot[2], Charlotte Bach[2], Michel Rivoire[4], Guillaume Passot[5], Thomas F. Baumert[2,6,7], Karim Majzoub[8], Eric Huntzinger[9], Bertrand Seraphin[9], David Durantel[1, $], Eloi R. Verrier[1,2,$] and Julie Lucifora[1,$]

[1]CIRI, Centre International de Recherche en Infectiologie, Univ Lyon, Inserm, U1111, Université Claude Bernard Lyon 1, CNRS, UMR5308, ENS de Lyon, F-69007, Lyon, France

[2]Université de Strasbourg, Inserm, UMRS 1110, Institute for Translational Medicine and Liver Disease, Strasbourg, France.

[3]INSERM, U1052, Cancer Research Center of Lyon (CRCL), University of Lyon (UCBL1), CNRS UMR_5286, Centre Léon Bérard, Lyon, France

[4]Centre Léon Bérard (CLB), INSERM, U1032, Lyon, France

[5]Service de chirurgie générale et oncologique, Hôpital Lyon Sud, Hospices Civils de Lyon Et CICLY, EA3738, Université Lyon 1

[6]Pôle Hépato-digestif, Service d'Hépato-gastroentérologie, Hôpitaux Universitaires de Strasbourg, Strasbourg, France.

[7]Institut universitaire de France (IUF), Paris, France.

[8]Institut de Génétique Moléculaire de Montpellier (IGMM), Université de Montpellier, CNRS UMR5535, Montpellier, France.

[9]IGBMC, Institut de Génétique et de Biologie Moléculaire et Cellulaire, CNRS UMR 7104, Inserm U 1258, Université de Strasbourg, Illkirch, France

* These three authors contributed equally as first authors

$ These three authors contributed equally as senior authors.

$ **Correspondence**. Dr. Julie Lucifora, julie.lucifora@inserm.fr; Dr Eloi R. Verrier, e.verrier@unistra.fr; Dr David Durantel, david.durantel@inserm.fr

Supplementary file

Methods

Cell culture and viral infections. HepaRG cells were cultured and differentiated as previously described (1, 2). Primary human hepatocytes (PHH) were isolated from human liver resections obtained from the "Centre Léon Bérard" (Lyon) and Hospital "Lyon Sud" with French ministerial authorizations (AC 2013-1871, DC 2013 – 1870, AFNOR NF 96 900 sept 2011) as previously described (3). HuH7.5-NTCP cells and HuH7-NTCP cells were cultured in 10% FCS-supplemented DMEM and differentiated with 2% of DMSO for 1 week. HBV (genotype D) inocula were prepared from HepAD38 supernatants (4) and used with a multiplicity of infection (m.o.i) of 500 vge/cells. HDV inocula (genotype 1) were prepared from supernatants from HuH7-2C8D as previously described (5) and used with a m.o.i of 10 vge/cells.

Plasmids, reagents and antibodies. IFN-α (Roferon) was purchased from Roche. The lentiviral vectors pLV[Exp]-Puro-CMV>HA/hHELZ2[NM_001037335.2] coding for HA-tagged (Nter) recombinant HELZ2β. Lentiviral particles were produced by the AniRA lentivector production facility from the CELPHEDIA Infrastructure and SFR Biosciences (UAR3444/CNRS, US8/Inserm, ENS de Lyon, UCBL) or in-house as described previously (6). peGFPC1-HELZ2, peGFPC1-HELZ2β plasmids were described in (7). Transfection of plasmids were performed with E5921 FuGENE® 4K Transfection reagent (Promega), according to manufacturer's recommendations. pSVLD3 plasmid allowing expression of the HDV genome (genotype 1, GenBank: M21012.1) was used to launch HDV replication, pcDNA3.1 plasmids with 2xRDeV and mmDeV genomes were transfected in cells as described in (8, 9) to launch RDeV and mmDeV replication. The following primary antibodies were used: anti-GFP (Abcam, ab290), anti-β-actin (Sigma, A2228), anti-HA (Sigma, H3663), β-tubulin (Abcam 6046), anti-RNA Pol II (Diagenode C1520004). The polyclonal rabbit antibody anti-HDAg was produced by Eurogentec for in-house use with the permission of Janssen (Belgium) and cross-reacted to recognize RDeV and mmDeV proteins. siRNA CTL (#D-001810-10-20) and siHDV (a combination of two siRNAs: AAGGAAGGCCCUCGAGAACAAUU, AAGAACCUCAGCAAGGAGGAAUU) were produced by Dharmacon. Cells were transfected with Lipofectamine RNAiMAX Transfection Reagent (ThermoFisher Scientific) according to the manufacturer's instructions.

Detection of proteins by western blot analyses. For analyses of total intracellular levels of proteins, cells were harvested in RIPA lysis buffer (Tris-HCl pH-7,5 10mM, NaCl 140mM, EDTA 1mM, EGTA 0,5mM, 1% Triton-X100, 0,1% SDS, 0,1% Na-Deoxycholate) containing protease inhibitors (Protein Cocktail Inhibitors from Sigma- Aldrich). Clarified lysates were subjected to SDS-PAGE and Western Blot transfer onto nitrocellulose membranes using the Trans-Blot® Turbo (BioRad) apparatus according to the manufacturer's instructions. For detection of (tagged) HELZ2 protein, proteins were separated on NuPAGE™ 3 à 8 %, Tris-acétate (Thermosficher) and the Western Blot transfer onto nitrocellulose membranes in wet tank Blotting systems (Mini Trans-Blot® Cell from BioRad) at 300mA constant amperage for 3h. Proteins were revealed by chemiluminescence (Super Signal West Dura Substrate, Pierce) using a secondary peroxydase-conjugated antibody (Dako) with Chemidoc MP System (BioRad).

Nucleic acid extractions, reverse transcription and qPCR/ddPCR analyses. Total intracellular RNAs were extracted with the "Monarch kit nucleic acid purification kit" according to the manufacturer's instructions (New England Biolabs). RNA reverse transcription was performed using the LunaScript RT Super Mix (New England Biolabs). Quantitative PCR for detection of all forms of HDV RNAs was performed using specific primers (Table 1) and normalized to GusB housekeeping mRNAs using then Luna Universal qPCR Master Mix (New England Biolabs).

RT-ddPCR for the specific quantification of HDV-G, HDV-AG and HDV mRNA. RNA reverse transcription was performed using the Lunascript RT master mix (New England Biolabs) with oligodT (for mRNA detection) or the following specific primers: 5'-GCTTCGAGCGGTAGGAGTAA-3' (for HDV-AG, named primer 4) and 5'-GATAAGCCTCACTCGTCCCC-3' (for HDV-G, named primer 5). ddPCR assay was performed using the "ddPCR Supermix for Probes (No dUTP)" (Bio-Rad) according to the manufacturer's instruction with specific primers (0,9 uM final) and probes (0,25 uM final) (Table 1).

Droplets were generated using and the" QX200™ Droplet Generator" (Bio-Rad) and analyzed after PCR with the "QX600 Droplet Reader" (Bio-Rad). Schematic overview of the assay is presented Figure 1B.

(m)RNAs	Forward primer sequence (5'-3')	Reverse primer sequence (3'-5')	Probe for ddPCR
Total HDV	CGGGCCGGCTACTCTTCT	AAGGAAGGCCCTCGAGAACA	
HDV (all RNA)	GTTGACCGAGGAAGACGAGA	CCTGCAGATTGGGGACGAA	[6-FAM]-AGAGTAGCCGGCCCGCC-[BHQ1]
HDV (G and AG)	GCTTCGAGCGGTAGGAGTAA	GATAAGCCTCACTCGTCCCC	[HEX]-ACGGGGCTAGCCGGTGG-[BHQ1]
HELZ2	GTGTCCTCCATCACCAAGAGC	TCCACAACGAAGCCCAGAAAC	
GusB	CCCACTCAGTAGCCAAGTCA	CACAAAACCCAGGCCAGAAA	[HEX]-CGTGTCCCTTCCTCCCCGAG-[BHQ1]

Table 1. Primers and probes for qPCR or ddPCR analyses.

Nuclear/cytoplasm separation and immunoprecipitation assays. Cells were used in native condition or chemically-cross-linked with 0,4% formaldehyde for 10min at RT, quenched with 0,25M glycine for 5min. Cells were mechanically lysed at 4°C in lysis buffer (PIPES 5mM, KCL 85mM, NP-40 0,5%) with protease inhibitors (Sigma-Aldrich). After low-speed centrifugation to separate nucleus from cytoplasm, nuclei were lysed in RIPA buffer. After sonication, both nucleus and cytoplasm clarified lysates were precleared with magnetic beads (DynabeadsTM Protein G, Thermo-Fisher) before immunoprecipitation with antibodies at 4°C. Magnetic beads were added for 2h to capture immune complexes. Flow-through (FT) fractions were collected, and beads were washed with four times with RIPA-buffer and once with TE (Tris-HCl pH-8 10mM, EDTA 10mM) and then incubated for 2h at 68°C in elution buffer (Tris-HCl pH-7,5 20mM; EDTA 5mM; NaCl 50mM; 1% SDS; Proteinase-K 50µg/mL). RNAs were extracted from the eluted immunoprecipitated materials using TRI Reagent™ (Thermo Fisher Scientific) and the levels of RNAs associated to the indicated proteins were analyzed by RT-ddPCR. 1:10 of FT from the no antibody controls were also subjected to the same RNA procedure and used as input for calculation of % of input.

Nascent RNA assay. HBV nascent RNAs were quantified as previously described (10, 11). Briefly, cells were incubated for 2h at 37°C with 5-bromouridine (BrU, Sigma), before RNA extraction and immunoprecipitation using an anti-BrdU antibody (BD Pharmingen) and Dynabeads coated with anti-mouse IgG (Life Technologies), overnight at 4°C. Captured RNAs were then eluted, concentrated (RNA Clean & Concentrator-25 w/ Zymo-Spin IIC Columns, Ozyme) and quantified by RT-qPCR. Total RNAs, extracted before IP were similarly quantified by RT-qPCR or RT-ddCPR. Controls were provided by cells treated with actinomycin D (Invitrogen, 1 µM; 1 h before labeling), or cells incubated in the absence of BrU.

RNA degradation assay. Cells were incubated for 24h at 37°C with 5-bromouridine (BrU, Sigma). Cells were washed extensively and lyzed at different time (chase). RNA extraction and immunoprecipitation using an anti-BrdU antibody (BD Pharmingen) and Dynabeads coated with anti-mouse IgG (Life Technologies) overnight at 4°C were performed. Captured RNAs were then eluted, concentrated (RNA Clean & Concentrator-25 w/ Zymo-Spin IIC Columns, Ozyme) and quantified by RT-qPCR or RT-ddCPR.

Data analysis. Statistical analyses were performed using GraphPad Prism software. The number of independent experiments and the number of biological replicates per experiment are indicated in the corresponding figure legends. Statistical comparisons were performed using all biological replicates across the experimental conditions. A non-parametric Mann–Whitney U test was used. Statistical significance was defined as follows: ****, $p < 0.0001$; ***, $0.0001 \le p < 0.001$; **, $0.001 \le p < 0.01$; *, $0.01 \le p < 0.05$.

Next-Generation Sequencing data. RNA-seq data presented in this study were previously published in (12) and accessible through Gene Expression Omnibus (GSE288203). Next-generation sequencing was performed by the Biomedical Sequencing Facility at CeMM Research Center for Molecular Medicine of the Austrian Academy of Sciences. For the data presentation of ISG expression, genes with an upregulation greater than 1 log2 fold change (log2FC) and an adjusted p-value < 0.05 upon IFN-α

treatment were considered ISGs. The expression of 150 ISGs commonly upregulated in both dHuH7.5-NTCP and dHepaRG cells was presented using Z-score transformation in Figure 2.

Transcriptomic analysis by digital multiplexed gene profiling using nCounter NanoString. Transcriptomic analyses using nCounter NanoString were performed according to manufacturer's instructions and as already described (13, 14). Specific probes for a set of 82 genes (listed in Table 2) were obtained from the manufacturer. Total RNA was extracted and subjected to NanoString nCounter Digital Analyzer system (Bruker, Billerica, Massachusetts, U.S). Raw data generated by the nCounter platform (NanoString Technologies) were processed and normalized using the nSolver Analysis Software (NanoString Technologies). Normalization was performed according to the manufacturer's guidelines, using a panel of five housekeeping genes (ATP5F1B, COX8A, HINT1, HNRNPA2B1 and NDUFA2) for biological normalization, along with the internal positive and negative controls included in the assay.

ADAR	NM_001111.3	6092-6191
AIM2	NM_004833.1	608-707
APOBEC3B	NM_004900.3	620-719
APOBEC3F	NM_145298.6	2337-2436
APOBEC3G	NM_021822.2	762-861
ATP5F1B	NM_001686.3	646-745
BAHCC1	NM_001080519.2	8109-8208
BATF2	NM_138456.3	2005-2104
BATF3	NM_018664.2	300-399
BST2	NM_004335.2	21-120
COX8A	NM_004074.2	116-215
DDX58	NM_014314.3	1390-1489
DDX60	NM_017631.5	661-760
DHX58	NM_024119.2	848-947
EBF4	NM_001110514.1	1998-2097
EIF2AK2	NM_001135652.2	517-616
ETV7	NM_001207035.1	577-676
GBP2	NM_004120.3	781-880
HELZ2	NM_033405.3	7299-7398
HES4	NM_001142467.1	558-657
HINT1	NM_005340.6	157-256
HNRNPA2B1	NM_002137.3	436-535
IDO1	NM_002164.5	370-469
IFI16	NM_001206567.1	667-766
IFI27	NM_005532.3	391-490
IFI35	NM_005533.3	416-515
IFIH1	NM_022168.2	186-285
IFIT1	NM_001548.3	1441-1540
IFIT2	NM_001547.4	1996-2095
IFIT3	NM_001031683.2	1471-1570
IFIT5	NM_012420.2	776-875
IFITM1	NM_003641.3	483-582
IFITM2	NM_006435.2	5-104
IFITM3	NM_021034.2	478-577
IRF1	NM_002198.2	16-115
IRF7	NM_001572.3	1764-1863
IRF9	NM_006084.4	386-485
ISG15	NM_005101.3	306-405

ISG20	NM_002201.5	865-964
KMO	NM_003679.3	596-695
MNDA	NM_002432.1	788-887
MOV10	NM_020963.3	421-520
MX1	NM_002462.2	1486-1585
MX2	NM_002463.1	2189-2288
NDUFA2	NM_002488.4	413-512
NMI	NM_004688.2	473-572
OAS1	NM_001032409.1	806-905
OAS2	NM_016817.2	481-580
OAS3	NM_006187.2	4981-5080
OASL	NM_198213.1	261-360
PATL2	NM_001145112.1	966-1065
PAX2	NM_000278.3	2862-2961
PML	NM_002675.3	282-381
PNPT1	NM_033109.4	563-662
POU3F2	NM_005604.2	3491-3590
PSMB8	NM_004159.4	1216-1315
RBM43	NM_198557.2	1231-1330
RORB	NM_006914.3	1841-1940
RPLP0	NM_001002.3	251-350
RSAD2	NM_080657.4	1038-1137
SAMHD1	NM_015474.2	641-740
SHFL	NM_001308277.1	331-430
SLFN11	NM_001104587.1	921-1020
SOCS1	NM_003745.1	1026-1125
SP100	NM_003113.3	912-1011
SP110	NM_004510.3	766-865
STAT1	NM_007315.3	1796-1895
STAT2	NM_005419.3	1391-1490
STING1	NM_198282.1	726-825
TBX1	NM_080646.1	541-640
TENT5A	NM_017633.2	3836-3935
TFEC	NM_012252.2	891-990
TRIM14	NM_014788.2	396-495
TRIM22	NM_006074.3	1746-1845
TRIM25	NM_005082.4	3415-3514
TRIM5	NM_033092.2	801-900
XAF1	NM_199139.2	314-413
XRN1	NM_019001.4	1239-1338
ZBP1	NM_001160419.2	681-780
ZFPM2	NM_012082.3	1851-1950
ZMYND15	NM_032265.1	1950-2049
ZNF107	NM_001013746.2	1217-1316

Table 2. Genes and probes' positions for Transcriptomic analyses using nCounter NanoString

References

1. Alfaiate D, Lucifora J, Abeywickrama-Samarakoon N, Michelet M, Testoni B, Cortay JC, Sureau C, Zoulim F, Deny P, Durantel D. 2016. HDV RNA replication is associated with HBV repression and interferon-stimulated genes induction in super-infected hepatocytes. Antiviral Res 136:19-31.
2. Gripon P, Rumin S, Urban S, Le Seyec J, Glaise D, Cannie I, Guyomard C, Lucas J, Trepo C, Guguen-Guillouzo C. 2002. Infection of a human hepatoma cell line by hepatitis B virus. Proc Natl Acad Sci U S A 99:15655-60.
3. Lecluyse EL, Alexandre E. 2010. Isolation and culture of primary hepatocytes from resected human liver tissue. Methods Mol Biol 640:57-82.
4. Ladner SK, Otto MJ, Barker CS, Zaifert K, Wang GH, Guo JT, Seeger C, King RW. 1997. Inducible expression of human hepatitis B virus (HBV) in stably transfected hepatoblastoma cells: a novel system for screening potential inhibitors of HBV replication. Antimicrob Agents Chemother 41:1715-20.
5. Bach C, Lucifora J, Delphin M, Heydmann L, Heuschkel MJ, Pons C, Goto K, Scheers E, Schuster C, Durantel D, Pauwels F, Baumert TF, Verrier ER. 2023. A stable hepatitis D virus-producing cell line for host target and drug discovery. Antiviral Res 209:105477.
6. Heuschkel MJ, Bach C, Meiss-Heydmann L, Gerges E, Felli E, Giannone F, Pessaux P, Schuster C, Lucifora J, Baumert TF, Verrier ER. 2024. JAK1 promotes HDV replication and is a potential target for antiviral therapy. J Hepatol 80:220-231.
7. Huntzinger E, Sinteff J, Morlet B, Seraphin B. 2023. HELZ2: a new, interferon-regulated, human 3'-5' exoribonuclease of the RNB family is expressed from a non-canonical initiation codon. Nucleic Acids Res 51:9279-9293.
8. Khalfi P, Denis Z, McKellar J, Merolla G, Chavey C, Ursic-Bedoya J, Soppa L, Szirovicza L, Hetzel U, Dufourt J, Leyrat C, Goldmann N, Goto K, Verrier E, Baumert TF, Glebe D, Courgnaud V, Gregoire D, Hepojoki J, Majzoub K. 2024. Comparative analysis of human, rodent and snake deltavirus replication. PLoS Pathog 20:e1012060.
9. Iwamoto M, Shibata Y, Kawasaki J, Kojima S, Li YT, Iwami S, Muramatsu M, Wu HL, Wada K, Tomonaga K, Watashi K, Horie M. 2021. Identification of novel avian and mammalian deltaviruses provides new insights into deltavirus evolution. Virus Evol 7:veab003.
10. Mueller H, Lopez A, Tropberger P, Wildum S, Schmaler J, Pedersen L, Han X, Wang Y, Ottosen S, Yang S, Young JAT, Javanbakht H. 2019. PAPD5/7 Are Host Factors That Are Required for Hepatitis B Virus RNA Stabilization. Hepatology 69:1398-1411.
11. Paulsen MT, Veloso A, Prasad J, Bedi K, Ljungman EA, Magnuson B, Wilson TE, Ljungman M. 2014. Use of Bru-Seq and BruChase-Seq for genome-wide assessment of the synthesis and stability of RNA. Methods 67:45-54.
12. Fouille R, Verrier ER, De Meyer A, Verhoye L, Michelet M, Barnault R, Pons C, Diaz O, Rivoire M, Passot G, Steinmann E, Wedemeyer H, Salvetti A, Pavio N, Doceul V, Darteil R, Meuleman P, Durantel D, Lucifora J. 2025. A novel in vitro system for simultaneous infections with hepatitis B, C, D and E viruses. JHEP Rep 7:101383.
13. Crouchet E, Bandiera S, Fujiwara N, Li S, El Saghire H, Fernandez-Vaquero M, Riedl T, Sun X, Hirschfield H, Juhling F, Zhu S, Roehlen N, Ponsolles C, Heydmann L, Saviano A, Qian T, Venkatesh A, Lupberger J, Verrier ER, Sojoodi M, Oudot MA, Duong FHT, Masia R, Wei L, Thumann C, Durand SC, Gonzalez-Motos V, Heide D, Hetzer J, Nakagawa S, Ono A, Song WM, Higashi T, Sanchez R, Kim RS, Bian CB, Kiani K, Croonenborghs T, Subramanian A, Chung RT, Straub BK, Schuppan D, Ankavay M, Cocquerel L, Schaeffer E, Goossens N, Koh AP, Mahajan M, Nair VD, Gunasekaran G, et al. 2021. A human liver cell-based system modeling a clinical prognostic liver signature for therapeutic discovery. Nat Commun 12:5525.

14. Verrier ER, Yim SA, Heydmann L, El Saghire H, Bach C, Turon-Lagot V, Mailly L, Durand SC, Lucifora J, Durantel D, Pessaux P, Manel N, Hirsch I, Zeisel MB, Pochet N, Schuster C, Baumert TF. 2018. Hepatitis B Virus Evasion From Cyclic Guanosine Monophosphate-Adenosine Monophosphate Synthase Sensing in Human Hepatocytes. Hepatology 68:1695-1709.

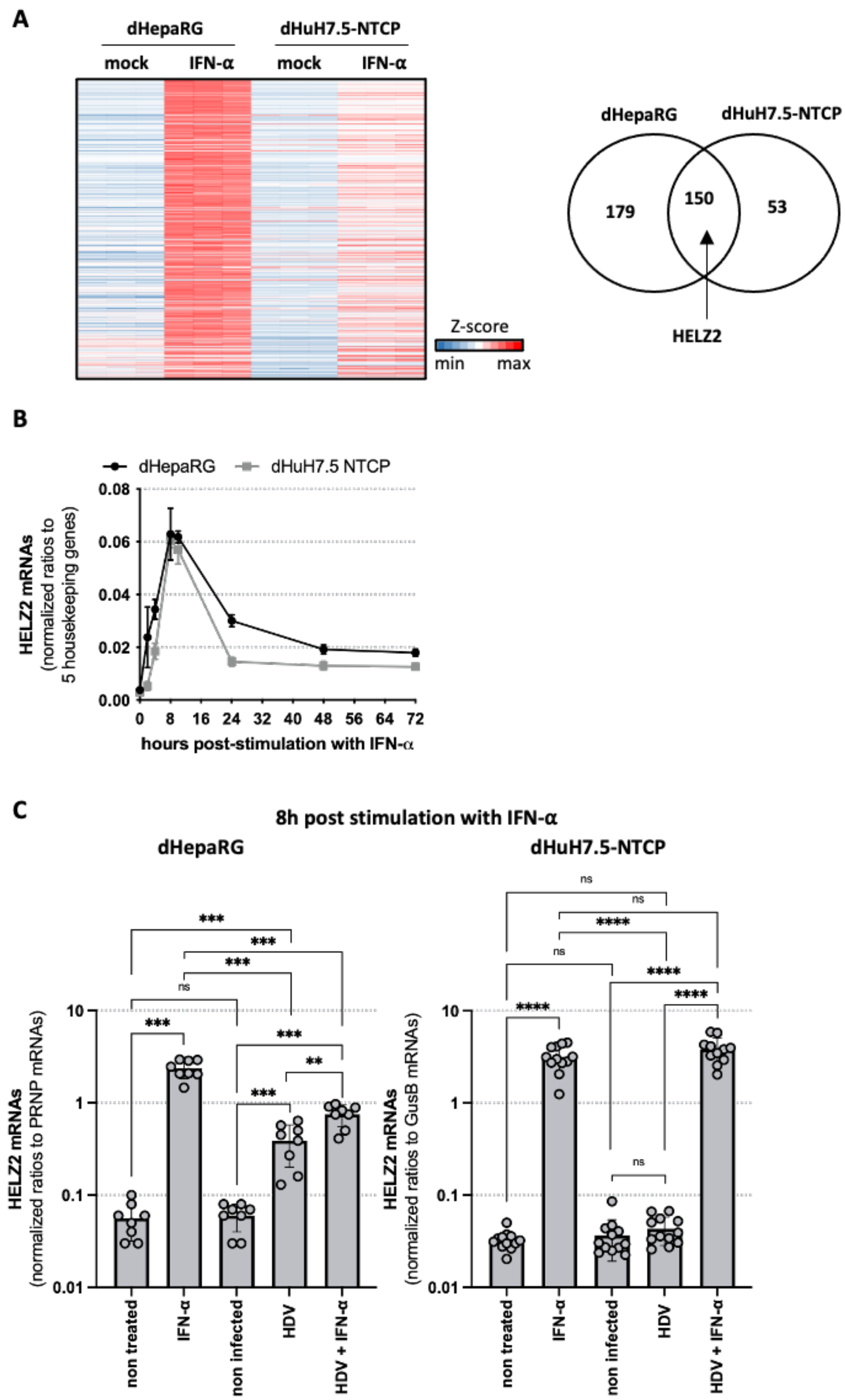

Figure S1: HELZ2 is an interferon stimulated gene induced upon IFN-α stimulation in both dHepaRG and dHuH7.5-NTCP cells. (A) dHepaRG and dHuH7.5-NTCP cells were stimulated with IFN-α (500 IU/mL) for 8h and total RNAs were extracted and subjected to RNA sequencing (GSE288203). Gene expression with Z-score transformation of 150 commonly upregulated genes in both cell lines is presented. Venn diagram (right panel) represents the number of genes upregulated in both cell lines upon IFN-α treatment, either commonly or specifically. (B) dHepaRG and dHuH7.5-NTCP cells were stimulated with IFN-α (500 IU/mL) and harvested at the indicated time points. Total RNAs were extracted and levels of *MX2* and *HELZ2* mRNAs analyzed by Nanostring assays. (C) dHepaRG or dHuH7.5-NTCP cells were infected or not with HDV and treated or not with IFN-α (500 IU/mL) for 8h 6

days post-infection. Cells were harvested and levels of intracellular *HELZ2* mRNAs were assessed by RT-qPCR.

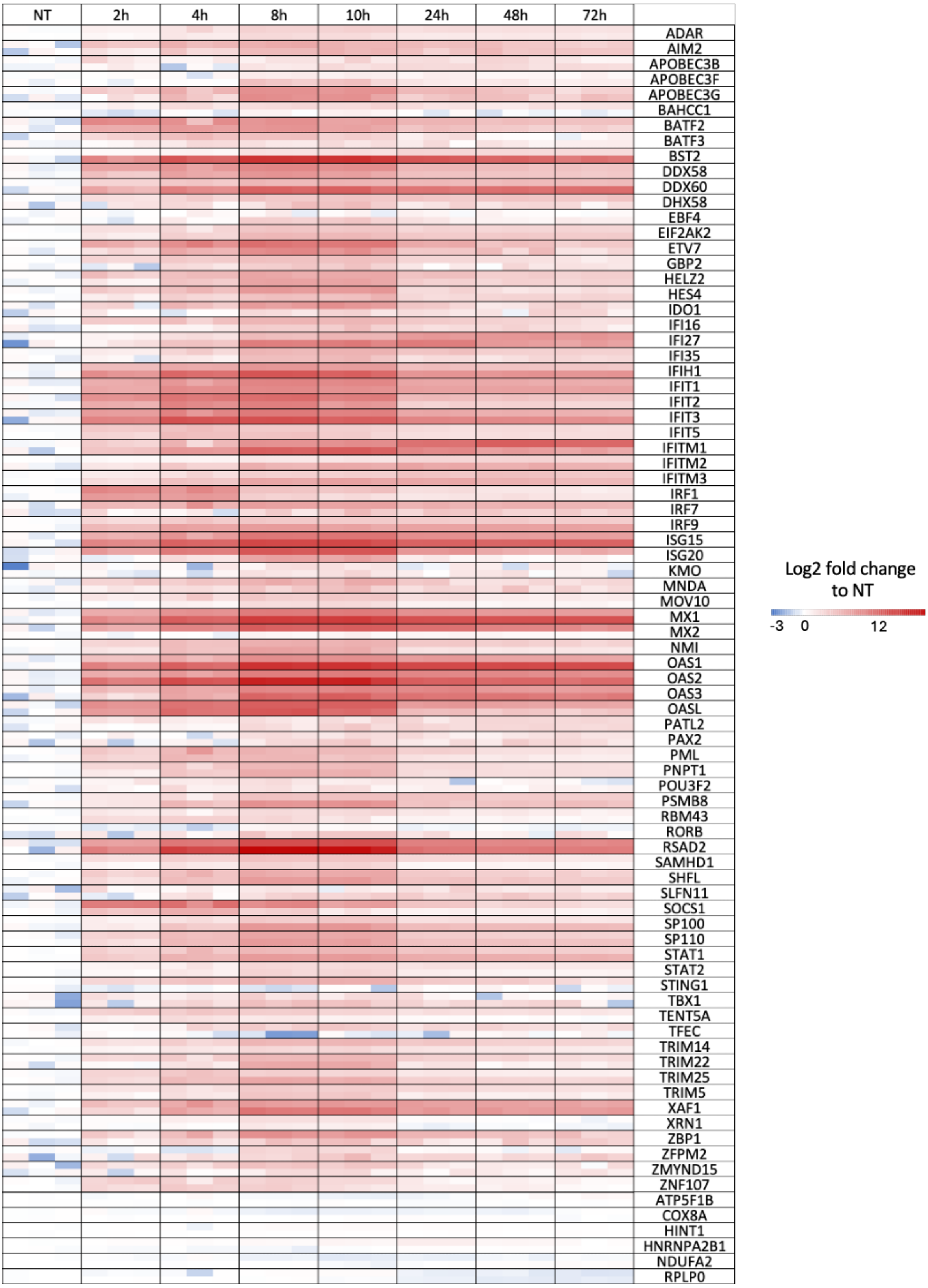

Figure S2. **Validation of ISG induction upon treatment with IFN-α.** dHepaRG and dHuH7.5-NTCP cells were stimulated with IFN-α (500 IU/mL) and harvested at the indicated time points. Total RNAs were extracted and levels of indicated mRNAs analyzed by Nanostring assays. 76 ISG mRNAs were tested (76 first raw) as well as five housekeeping mRNAs (5 last raw). Data are expressed as Log2 fold change

compared to non-treated cells (NT) for each mRNAs. Results from triplicate are shown and top lines represented dHepaRG and bottom line dHuH7.5-NTCP cells for each gene

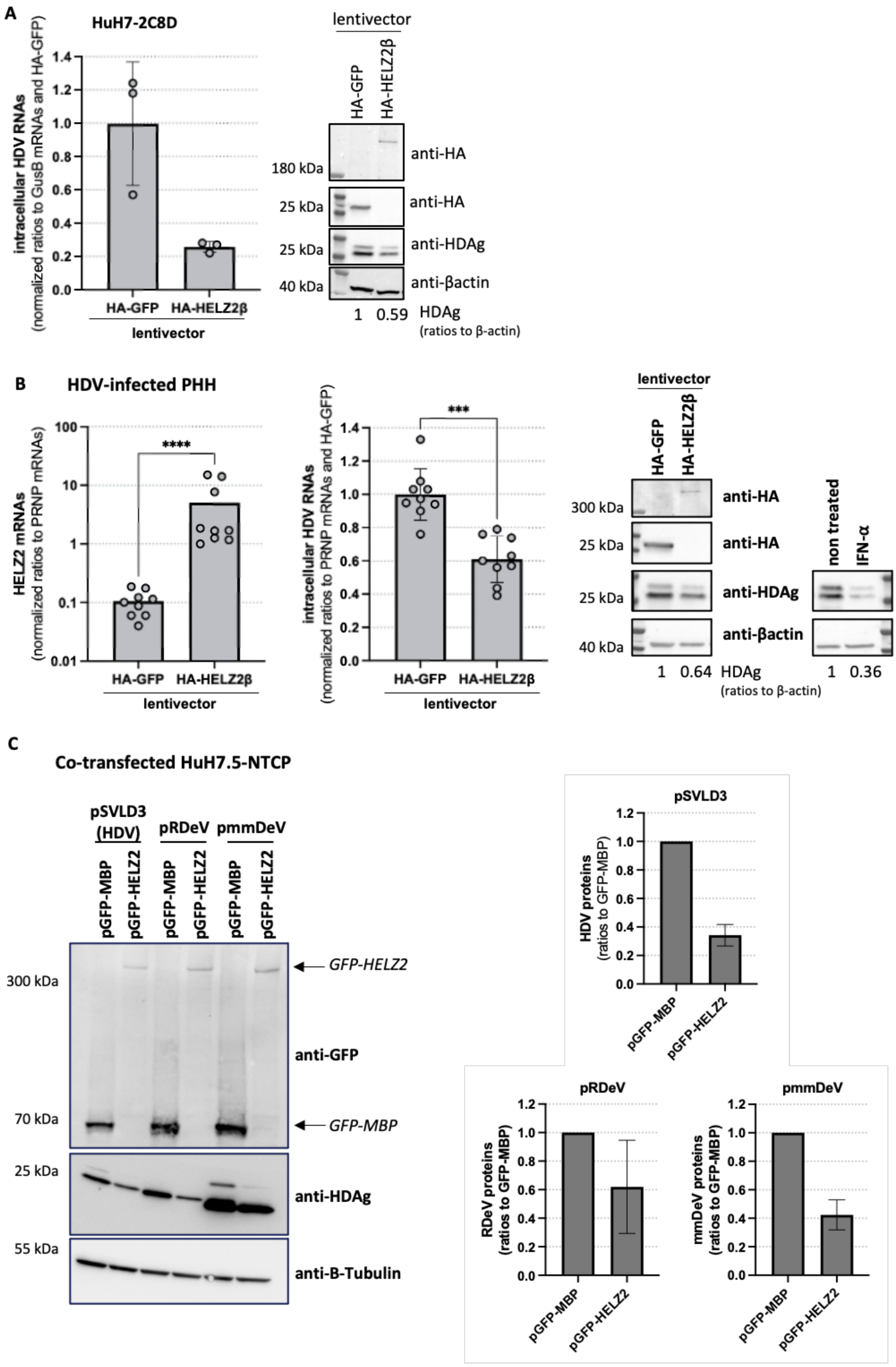

Figure S3. **Over-expression of HELZ2 decreased levels of intracellular HDV and DeV replication markers.** (A) HuH7-2C8D cells were transduced with lentivectors encoding HA-tagged version of GFP or HELZ2β (1 vge/cell). At 6 post transduction, cells were lysed and levels of total intracellular HDV RNAs and proteins analyzed by RT-qPCR and by western blotting respectively. Bars are the means ± SD of one experiment performed with three biological replicates. Dots represent individual replicates. (B) PHH were infected with HDV (10 vge/cells) and transduced 3 days later with lentivectors encoding for an HA-tagged version of GFP or HELZ2β (1 vge/cell). At 10 dpi, intracellular RNAs were extracted and

levels of total HDV RNAs and *HELZ2* mRNAs analyzed by RT-qPCR. Levels of indicated proteins were analyzed by western blotting. Bars are the means ± SD of three independent experiments each performed with three biological replicates. Dots represent individual biological replicates. As control, PHH were treated with IFN-α (500 IU/mL) from day 3 to day 10 post-infection with HDV and levels of HDV proteins assessed by western blot analyses. (C) HuH7.5-NTCP cells were co-transfected with pSVLD3, pRDeV or pmmDeV and plasmids encoding a GFP-tagged version of HELZ2 or MBP (control protein). At day 6 post-transfection, cells were lysed and levels of GFP-MBP, GFP-HELZ2, HDV, RDeV and mmDeV proteins were analyzed by western blot. Representative blots from one experiment are shown. Graphs show the mean ± SEM of the quantification of blots from four independent experiments.

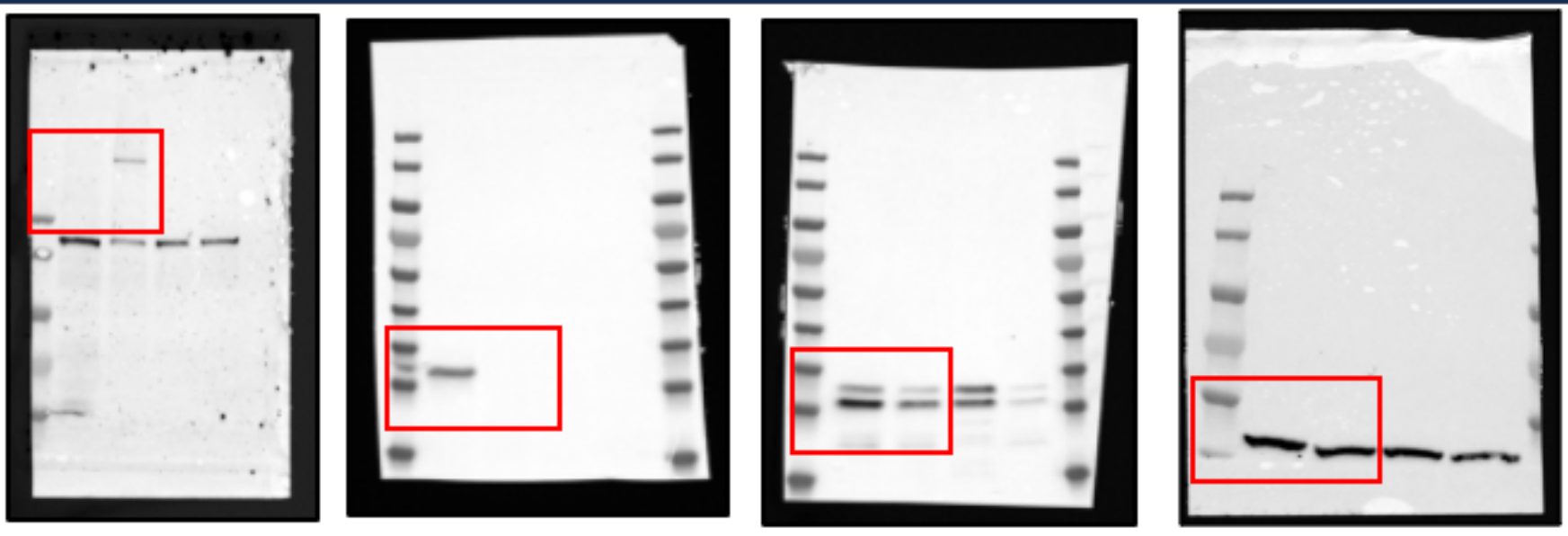

Figure S3A

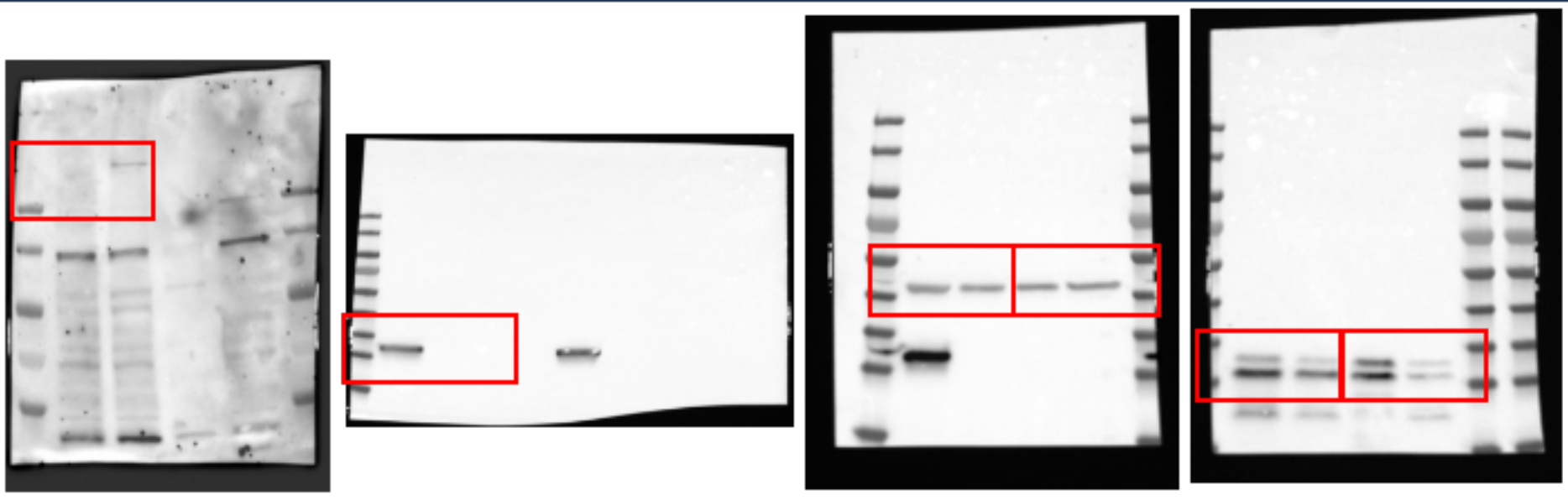

Figure S3B

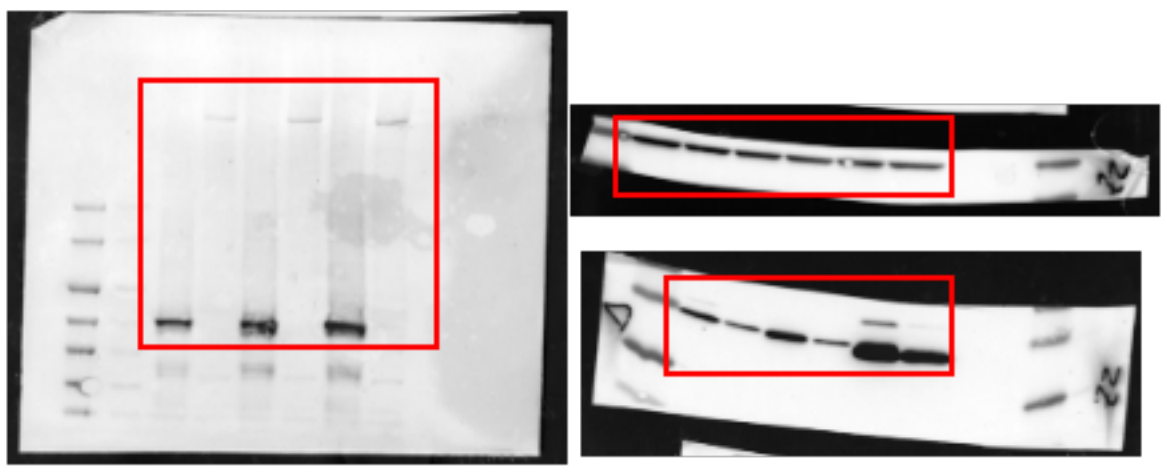

Figure S3C

Figure S4. Full blots
